# Magnetic Resonance Imaging of Endometriosis: The Role of Advanced Techniques

**DOI:** 10.3390/jcm13195783

**Published:** 2024-09-28

**Authors:** Laura Alonzo, Roberto Cannella, Giuseppe Gullo, Giulia Piombo, Giuseppe Cicero, Alessandra Lopez, Valentina Billone, Alessandra Andrisani, Gaspare Cucinella, Antonio Lo Casto, Giuseppe Lo Re

**Affiliations:** 1 Department of Biomedicine, Neuroscience and Advanced Diagnostic (BI.N.D.), University of Palermo, 90127 Palermo, Italy; laura.alonzo308@gmail.com (L.A.); piombogiulia92@gmail.com (G.P.); antonio.locasto@unipa.it (A.L.C.); giuseppe.lore01@unipa.it (G.L.R.); 2 Unit of Obstetrics and Gynecology, AOOR Villa Sofia Cervello, University of Palermo, 90100 Palermo, Italy; gullogiuseppe@libero.it (G.G.); alessandralopez91@gmail.com (A.L.); valentina.billone@gmail.com (V.B.); gaspare.cucinella@unipa.it (G.C.); 3 Department of Precision Medicine in Medical, Surgical and Critical Care Area, University of Palermo, 90127 Palermo, Italy; giuseppe.cicero@unipa.it; 4 Unit of Gynecology and Obstetrics, Department of Women and Children’s Health, University of Padua, 35128 Padua, Italy; alessandra.andrisani@unipd.it

**Keywords:** female pelvis, endometriosis, magnetic resonance imaging, diffusion weighted imaging, tractography

## Abstract

Endometriosis is a chronic inflammatory disease that affects about 10% of women, and it is characterized by the presence of endometrial tissue outside the uterine cavity. Associated symptoms are dyspareunia, chronic pelvic pain, and infertility. The diagnosis of endometriosis can be challenging due to various clinical and imaging presentations. Laparoscopy is the gold standard for the diagnosis, but it is an invasive procedure. The literature has increasingly promoted a switch to less invasive imaging techniques, such as ultrasound and magnetic resonance imaging (MRI). The latter, also in relation to the latest technological advances, allows a comprehensive and accurate assessment of the pelvis and it can also identify sites of endometriosis that escape laparoscopic evaluation. Furthermore, MRI has been found to be more accurate than other imaging techniques in relation to its improved sensitivity and specificity in identifying disease sites, also due to the role of new emerging sequences. This article aims to review the current role of advanced MRI applications in the assessment of endometriosis.

## 1. Introduction

Endometriosis is a chronic, estrogen-dependent, inflammatory disease affecting women of reproductive age. It is characterized by the presence of endometrial tissue outside its usual location. This tissue, heterotopia, consisting of stroma and glands, responds functionally to the same hormones that act on the normal uterine mucosa during the menstrual cycle in terms of proliferation, differentiation, and bleeding. Depending on the location of the ectopic endometrial tissue, a distinction is made between an external form, characterized by the presence of endometrial tissue outside the uterus, and an internal form, also known as adenomyosis, with endometrial tissue in the thickness of the uterine myometrium. External pelvic endometriosis most commonly involves the ovaries, rectovaginal septum, uterosacral ligaments, and Douglas pouch. Deep pelvic endometriosis (DPE) occurs if the endometrial tissue infiltrates the peritoneum for more than 5 mm. In some cases, organs such as bowel loops and the urinary tract—or more rarely, distant sites such as the diaphragm, pericardium, pleura, or brain—may be affected, giving the definition of extragenital endometriosis.

Endometriosis can cause various symptoms, including chronic pelvic pain, painful menstruation, and fertility problems, and it can significantly affect a woman’s quality of life [1,2]. The heterogeneity of symptoms in patients with endometriosis has further complicated efforts to address this condition. Laparoscopy is still considered the gold standard in endometriosis diagnosis, but it is an invasive procedure with a risk of complications and false negative results [3]. There is increasing support for the clinical value of noninvasive imaging techniques, particularly ultrasound (US) and magnetic resonance imaging (MRI), in the diagnosis of endometriosis [4].

Transvaginal US is considered the initial modality to image endometriosis, due to its availability and low cost. However, MRI surpasses US in terms of the diagnosis and characterization of endometriotic lesions, thanks to its superior soft tissue resolution, improved reproducibility, and visualization of a larger pelvic volume. MRI’s multiplanar imaging capability, including non-orthogonal views, further enhances its diagnostic value together with its ability to differentiate tissue types and its sensitivity to flowing blood [5].

In 2017, the European Society of Urogenital Radiology (ESUR) published guidelines that recommend MRI as a second-line technique after transvaginal US for evaluating endometriosis [6]. ESUR also suggests using MRI for preoperative staging when transvaginal US results are equivocal or when a symptomatic patient has a negative transvaginal US [6].

There is considerable variability in MRI protocols used for studying endometriosis. The majority of published studies employ a 1.5 T or 3.0 T scanner and high-resolution phased array coils (with 8–16 channels) [6]. Operating at 3.0 T yields high-spatial-resolution images and accurately depicts all locations of DPE. While standardized MRI protocols have been widely adopted in clinical practice, new MRI techniques and applications are emerging for the evaluation of endometriosis, such as susceptibility-weighted imaging (SWI) and tractography, while the role of other sequences such as diffusion-weighted imaging (DWI) and post-contrast sequences is still debated [6].

This article aims to review the current role of advanced MRI sequences and applications in the assessment of endometriosis.

## 2. MRI Findings of Pelvic Endometriosis

Pelvic MRI can be performed regardless of the phase of the menstrual cycle. A moderate degree of bladder repletion is required for the proper evaluation of the anterior pelvic compartment. Vaginal and rectal filling with gel remains optional [6]. The administration of antiperistaltic agents may instead be helpful, mainly in evaluating adenomyosis, by reducing uterine contractions [7]. On the other hand, in suspicion of small bowel involvement, MR enterography, i.e., an MRI technique for the diagnosis of small bowel disorders, has shown high diagnostic accuracy [8].

The complexity of endometriosis arises from the fact that it can manifest with different subtypes, including superficial (Sampson’s disease), endometrioma, and DPE. It can also be either pelvic or extra-pelvic. Among these types, the most challenging to diagnose by MRI are implants on the peritoneal surface that measure only millimeters in size and can also be easily missed during laparoscopy [9]. If these implants hold hemorrhagic contents, appearing as hyperintense foci on T1-weighted images with fat suppression, they can be identified by MRI [6].

The accuracy of MRI may vary depending on the experience of the radiologists. Studies have indirectly investigated the role of radiologists’ experience in detecting endometriosis on MRI by including readers with different levels of expertise. Only one study, conducted by Bruyère et al. [10], specifically examined how radiologists’ experience levels affect the diagnosis of endometriosis on MRI. The study revealed that, while radiologists with varying levels of experience exhibited similar performance in identifying and characterizing endometriomas, there was significant variability in the interpretation of DPE, particularly when it was located in the posterior compartment. This suggests that patients with a clinical suspicion of endometriosis should be referred to radiological centers with the appropriate expertise [10].

For detecting DPE, T2-weighted sequences without fat suppression are considered the most effective sequences [6]. Fat-saturated T2-weighted sequences should not be used in a protocol for the study of endometriosis as the recognition of endometriosis foci is based on the contrast between the high signal intensity of the fat and the low signal intensity of the endometriosis nodules [7]. According to ESUR guidelines [6], the MRI protocol should include at least two orthogonal thin-section T2-weighted image planes (sagittal and axial). The acquisition of oblique planes has proven useful, especially for visualizing specific anatomical structures, such as the uterosacral ligaments, which are a frequent site of DPE [11]. Other studies have also highlighted the potential value of 3D-T2 sequences in evaluating DPE [12].

For evaluating adnexal endometriosis, T1-weighted sequences without and with fat suppression are mandatory. Other fat suppression techniques, such as STIR, can lead to incorrect diagnoses and should not be used for assessing adnexal lesions [7].

### 2.1. Endometrioma

The most common site for the abnormal implantation of endometrial tissue outside the uterus is the ovary, where repeated internal bleeding can lead to the formation of a large hemorrhagic cavity known as an endometrioma.

**Figure 1:**
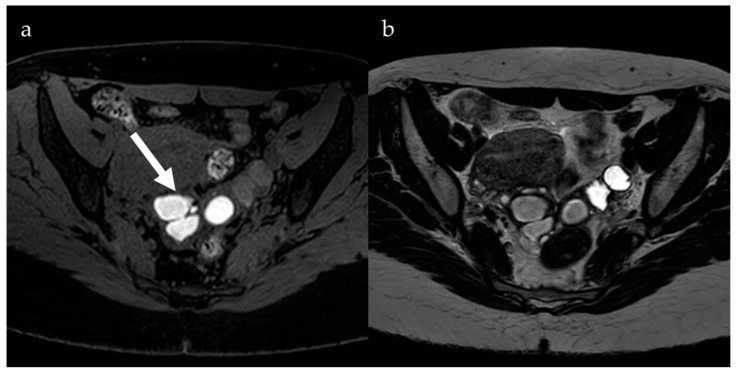
Right and left ovarian cysts showing high signal content on an axial fat-saturated T1-weighted image (**a**, arrow) and low signal on an axial T2-weighted image (**b**), suggesting hemorrhagic content. Both ovaries appear enlarged and in close relation, located in the rectouterine space (“kissing ovaries”).

Although it is possible to diagnose endometrioma with US, MRI is preferred for its greater specificity [13]. In addition, endometrioma is frequently associated with the presence of deep endometriosis, which is better evaluated with MRI. Imaging features for diagnosing ovarian endometriotic cysts include bilateral and well-defined multilocular cysts with low-intensity walls due to hemosiderin deposits on T1- and T2-weighted images and hyperintense content on T1-weighted sequences [14]. The presence of multiple hyperintense cysts on T1-weighted images, known as “multiplicity”, is a characteristic finding of repeated bleeding and the formation of new blood locules (Figure 1). These cysts do not show signal suppression on T1-weighted fat-suppressed sequences, resulting in a finding referred to as “light bulb bright” [14].

T2-shading, i.e., decreased signal content with respect to T1, which reflects chronic bleeding with high levels of iron and protein, and the “T2 dark spot sign”, together with adhesion to surrounding anatomical structures, are also high-sensitivity features in the diagnosis of endometrioma [13,14]. However, it is important to note that low intensity on T2-weighted images can also be observed in other cystic lesions, such as hemorrhagic functional cysts or ovarian tumors. In that case, the “T2 dark spot sign” is an important MRI finding with high specificity (93%) for diagnosing ovarian endometriotic cysts [15]. T2 dark spots are well-defined hypointense foci within the cyst on T2-weighted images, different in shape, and are believed to represent the presence of chronic retracted blood clots [15].

Another finding associated with endometriosis is hematosalpinx. The presence of a dilated Fallopian tube with hyperintense signal on T1-weighted images is suggestive of hematosalpinx. The inner serpiginous morphology or incomplete septa can usually help identify the origin from the Fallopian tube and differentiate hematosalpinx from endometrioma [13].

### 2.2. Deep Pelvic Endometriosis

**Figure 2:**
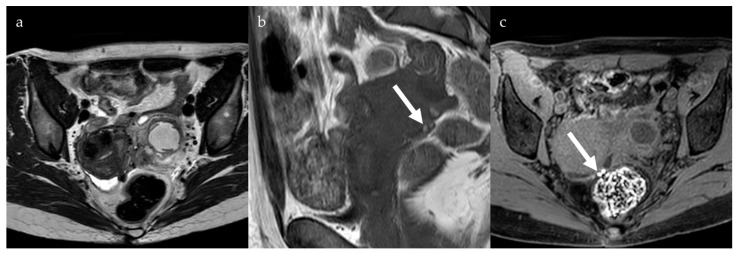
Hypointense nodules in the pouch of Douglas on an axial T2-weighted image (**a**) with some small high-signal foci on a coronal T1-weighted image (**b**, arrow), more evident on an axial fat-saturated T1-weighted image (**c**, arrow), compatible with deep pelvic endometriosis with evidence of bleeding.

DPE is defined as endometriosis that infiltrates the peritoneum to a depth of more than 5 mm. On MRI, DPE has non-specific signal patterns, with hypointense nodular lesions or soft tissue thickening with irregular, indistinct, or stellate margins on both T1- and T2-weighted images (Figure 2). Occasionally, hyperintense lesions on T1-weighted images, especially on fat-saturated sequences, can be observed, indicating hemorrhagic foci. The diagnostic performance of MRI for the detection of DPE is relatively high, with a sensitivity of 90% and specificity of 91% [16].

Attention should be given to hypointense tissue thickening or retractions in the following common locations: the posterior side of the uterus, retrocervical area, uterosacral ligaments, posterior fornix of the vagina, rectovaginal septum, and anterior side of the rectosigmoid. The presence of hemorrhagic foci facilitates and confirms the diagnosis, but any hypointense nodular or plaque-like thickening of these structures on T2-weighted images should raise suspicion.

Regarding the posterior compartment of the pelvis, the following MRI findings were reported: a retroflexed uterus, an elevation of the posterior vaginal fornix, the intestinal adherences, faint strands between the uterus and intestine, and fibrotic nodules covering the surface of the uterus. Macario et al. reported that, among these findings, a retrouterine fibrous mass, intraperitoneal fluid displacement, and adherence of bowel loops showed the highest performances (92.8%, 93.1%, and 86.1% accuracy, respectively) [17].

MRI has been shown to have superior diagnostic capability in detecting endometriosis in the utero-sacral ligaments [18]. MRI findings of rectal endometriosis are the “fan-shaped” and “mushroom cap” appearances. The presence of these signs suggests muscular invasion by endometrial tissue [19]. A rectal circumferential involvement greater than 135° and a lesion thickness greater than 14 mm are specific predictors for segmental resection in rectal endometriosis [20].

On MRI, bladder endometriosis appears as hypointense nodular thickening on both T1- and T2-weighted images, and there may be high signal foci indicating ectopic endometrial glands and hemorrhage [21].

### 2.3. Extra-Pelvic Endometriosis

Among extra-pelvic sites, the abdominal wall and thoracic region are most commonly affected [22]. Regarding the abdominal wall, surgical scars (such as those from a C-section), the umbilical region, and the inguinal canal are frequently involved [22]. While US may be useful due to the superficial nature of these last lesions, MRI allows for a more specific diagnosis as it can detect small hyperintense foci on T1-weighted images, distinguishing endometriotic implants from hypertrophic scars or desmoid tumors. In diaphragmatic endometriosis, MRI has a sensitivity of between 78 and 83% [23].

## 3. Role of Contrast Agents

Prior studies have reported contradictory results on the utility of intravenous gadolinium administration in various locations of DPE [24,25,26,27]. However, gadolinium contrast administration is suggested as an option in the evaluation of indeterminate adnexal endometriosis [6].

**Figure 3:**
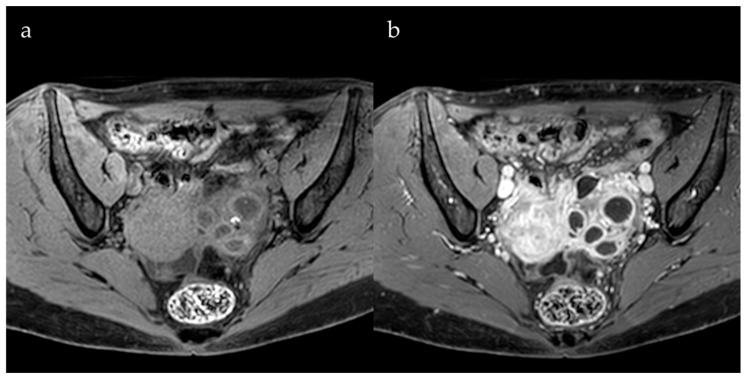
Axial pre-contrast (**a**) and axial post-contrast (**b**) images demonstrate a left pyosalpinx with an enlarged and multicystic ovary attributable to an ovarian tube abscess, both demonstrating wall enhancement.

Gadolinium contrast agents can aid in distinguishing endometrial cysts from other adnexal lesions, for example, an endometrioma from a tubo-ovarian abscess or another hemorrhagic cyst, particularly when intense wall enhancement is observed (Figure 3).

Furthermore, endometriotic cysts carry a potential risk of malignant transformation. MRI findings include the presence of mural nodules with enhancement and the increased size of the lesion. A diameter greater than 9 cm is indicated to be an independent risk factor for ovarian cancer [28]. A less specific sign is the disappearance of T2 shading, potentially correlated with secretions produced by the neoplastic tissue leading to the dilution of the hemorrhagic fluid [29].

## 4. Diffusion-Weighted Imaging

DWI is an established sequence in the gynecological MRI protocol, in addition to the conventional MRI sequences.

DWI is an echoplanar imaging (EPI) sequence that allows the measurements of the random movements of water molecules in biological tissues (also known as Brownian movement). The free diffusion of water molecules can be restricted depending on tissue type, intra-cellular structure, and specific pathologies (e.g., inflammation, ischemia, increased cellularity). The more restricted the movement of water, the higher the signal intensity generated on DWI.

Diffusion-weighted images are acquired with a series of different strong magnetic field gradients, referred to as b-values. The acquisition of a minimum of two b-values allows for the generation of the apparent diffusion coefficient (ADC) map. The ADC map provides a quantitative assessment of the degree of diffusion and restricted diffusion is displayed as low signal intensity on the ADC map.

Malignant neoplasms, but also some hypercellular tissues, can demonstrate restricted diffusion including the endometrium in secretory-phase, lymph nodes, and cystic lesions with high-protein content [30,31].

DWI proved to be valuable in evaluating various gynecological diseases, especially in malignancies and endometriotic lesions, which exhibit restricted diffusion.

**Figure 4:**
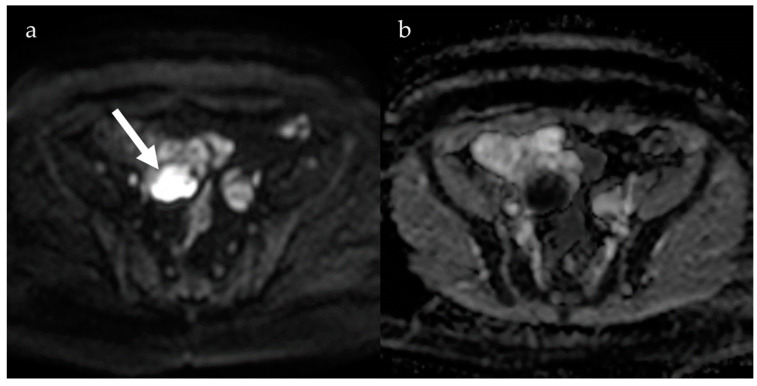
Diffusion-weighted imaging at b = 800 (**a**) shows a hyperintense lesion (arrow) with hypointensity on ADC map (**b**) indicating restricted diffusion, consistent with endometrioma.

DWI could help in distinguishing between DPE and other gynecological conditions. Endometrial cysts usually have a significantly lower ADC than functional ovarian cysts (Figure 4), and shading on T2-weighted images is in a linear relationship with ADC values [32,33]. In contrast, Moteki et al. found a weak correlation between ADC values and T2 signal in endometrial cysts because of independence from methemoglobin-related paramagnetic effects [34]. However, ADC values have about the same ability to differentiate endometrial from other pelvic cysts as does signal intensity on T1- and T2-weighted images [32,33,34].

Furthermore, cystic components of endometrial cysts and malignant ovarian cystic tumors have lower ADC values compared to other benign ovarian cysts. Therefore, DWI may not be suitable for differentiating between benign and malignant lesions [31,33].

During pregnancy, endometriotic cysts exhibiting transient decidualization can mimic ovarian tumors on US and MRI. DWI and ADC maps can help in the diagnosis. Endometrial tissue shows a consistently high signal intensity on both DWI and ADC and has a signal intensity very similar to placental tissue on T2-weighted imaging, usually having a large nodule implantation base [35].

Finally, Singh et al. highlighted DWI’s usefulness in a case of subdiaphragmatic endometriotic lesion [36].

## 5. Susceptibility-Weighted Imaging

SWI is a relatively novel high-spatial-resolution MRI sequence that has recently been applied in abdominal imaging for the detection of paramagnetic deposits, including blood. This sequence combines magnitude and phase information to enhance the susceptibility differences between tissues. Compared to the conventional gradient echo sequences, SWI is a more sensitive technique for the detection of small bleeding, blood products, and iron deposits.

Although SWI is mainly acquired for neuroimaging examinations for detecting microhemorrhages, this sequence has been recently applied in abdominal imaging [37].

Some studies investigated its role in the assessment of deep infiltrating endometriosis related to its higher sensitivity to blood products compared to conventional MRI sequences [38,39].

Endometriotic lesions are subject to repeated cycles of bleeding and may contain blood products at every stage [40,41]. Studies aimed at evaluating the diagnostic accuracy of these sequences agreed that the inclusion of SWI within the standard MRI protocol corresponded to an increase in the sensitivity to the detection of deep endometriotic lesions [37].

However, the sensitivity in the detection of superficial endometriotic lesions was not significantly increased [42] and it could also lead to a numerical and dimensional overestimation [43]. Susceptibility artifacts caused by intestinal gas are a disadvantage of this sequence, especially in the pelvic region.

## 6. Magnetic Resonance Neurography and Diffusion Tensor Imaging

A rare but important extra-pelvic endometriosis manifestation is represented by the compression or infiltration of nerves near the lesions causing irritation and pain [44].

Intra-pelvic nerve entrapments cause sciatica; pudendal neuralgia; and urinary, anorectal; and sexual dysfunction, which can be debilitating and significantly impact one’s quality of life [45]. Imaging modalities are a fundamental noninvasive exam in the diagnosis of pelvic nerve endometriosis as well as its preoperative planning [46].

**Figure 5:**
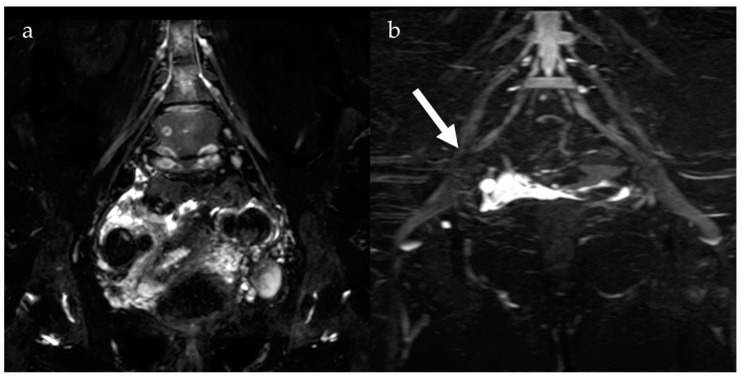
Example of 3D MR neurography showing lumbosacral plexus nerve roots. Images are displayed in the coronal plane. In a healthy woman (**a**), the fiber bundles display a homogeneous appearance and regular course bilaterally. In a woman affected by endometriosis (**b**), the fiber bundles display a discontinuous and partially interrupted course on the right side (arrow).

MR neurography (MRN) includes 3D, high-spatial-resolution, isotropic, fat-suppressed (with inversion recovery or Dixon technique) sequences, with the possibility of multiplanar reconstruction, and it is currently used to support the diagnosis of extraspinal lumbosacral plexus entrapments. However, for the intra-pelvic portions of the lumbosacral plexus, its accuracy remains limited [47]. MRN looks for asymmetries in the signal and morphology (thickening, narrowing, or trajectory deviations) of the intra-pelvic nerve bundles (Figure 5).

Other MRI findings in addition to those suggestive of DPE are asymmetry between the internal iliac arteries (possible cause of neurovascular conflict), adhesions, fibrosis, anomalous bundles of the piriformis muscle originating medial to the sacral foramina, and tumors of the nerve sheath, which are possible differential or exclusion diagnosis.

The relationship between endometriosis and pain is not attributable only to the presence of ectopic lesions but also to how these lesions, which undergo a process of neurogenesis, sensitize the central nervous system [48]. In the process of neurogenesis, the fibers sprout in a disorganized manner and it is likely that this disorganization also affects the lumbosacral roots in their most proximal segments [47].

Diffusion tensor imaging (DTI) and tractography represent innovative diagnostic tools for better assessing pelvic pain in patients suffering from endometriosis since they can obtain both “structural” and “ultrastructural” information about peripheral nerves and spinal roots. DTI assesses the integrity of fiber tracts by measuring the fractional anisotropy [49]. DTI allows for the identification of the anisotropic character of water diffusion processes in biological tissues with a high number of fibers, such as muscles or white matter in the nervous system, by tracing the diffusion tensor maps. Since diffusion occurs in three-dimensional structures, molecular mobility in tissues is not the same in every direction; this anisotropy property is related to the presence of obstacles that limit molecular motions in some directions. Since water tends to diffuse in the fibrous tissues (especially in the white matter) following the orientation of the fibers, DTI becomes an indicator of the functional organization, allowing the identification of the mutual connections between the different functional centers and highlighting alterations related to pathological situations [50].

**Figure 6:**
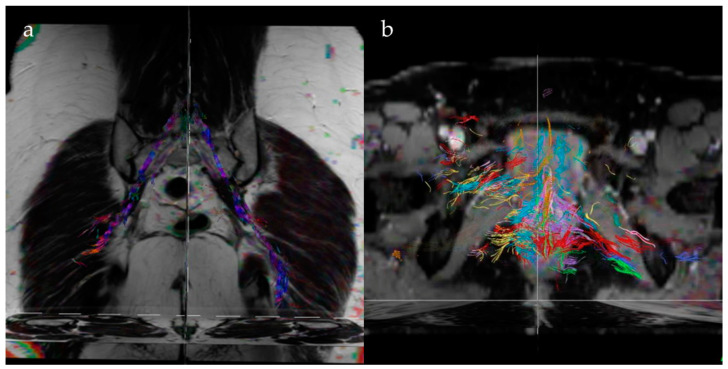
Fiber tracking reconstruction in two women affected by endometriosis of the posterior compartment (**a**,**b**). The fiber bundles in both cases are short, stubby, and have lots of branches.

Tractography imaging provides a colored visual representation of neural traits, using data collected from DTI (Figure 6). The color code is codified as follows: red for transverse, green for anteroposterior, and blue for craniocaudal fibers. The fibers with an oblique orientation are represented with colors deriving from the combination of the three primary colors: magenta (deriving from the bluest red), yellow (deriving from the reddest green), and cyan (deriving from the bluest green).

In a sample of 30 women suffering from chronic pelvic pain diagnosed with endometriosis, Manganaro et al. demonstrated a correlation between the reported symptoms and the disorganized appearance of the fibers of the nerve roots of S1, S2, and S3 compared to the control group, with a value of FA significantly reduced [47]. Porpora et al. also showed how diffusion tensor evaluation provides results that correlate with the severity of dysmenorrhea, pain duration, the presence of tubo-ovarian and cul-de-sac adhesions, and DPE, helping in the formulation of the correct therapeutic plan [51].

## 7. Conclusions

MRI is widely applied in the diagnosis of endometriosis, providing the accurate detection and characterization of the endometrial lesions. Future studies and meta-analyses are needed to improve the evidence supporting the clinical use of advanced MRI techniques. New MRI sequences and protocols could enhance the diagnostic capability of MRI, and they have the potential to improve the management of women affected by endometriosis.

## Data Availability

Not applicable.
